# Red algae natural products for prevention of lipopolysaccharides (LPS)-induced liver and kidney inflammation and injuries

**DOI:** 10.1042/BSR20202022

**Published:** 2021-01-15

**Authors:** Asmaa Nabil-Adam, Mohamed A. Shreadah

**Affiliations:** Marine Biotechnology and Natural Products Laboratory, National Institute of Oceanography and Fisheries, Cairo, Egypt

**Keywords:** G.Oblongata red algae, inflammation, kidney, liver, LPS, natural products

## Abstract

**Background:** The liver and kidney inflammation due to bacterial infection is one of the most common pathological problems leading to tissue damage or disease. In many liver and kidney disorders, which represent serious global health burden with a high economic cost, oxidative stress-related inflammation and apoptosis are important pathogenic components, finally resulting in acute liver and/or kidney failure. Erythropoietin and its analogues are well known to influence the interaction between apoptosis and inflammation in liver and kidney. **Objective**: The aim of the present study is to investigate and clarify the effect of *Galaxaura oblongata (G. oblongata)* red algae on lipopolysaccharides (LPS)-induced acute liver and kidney injury of mice with endotoxemia and associated molecular mechanism from inflammation, apoptosis and oxidative stress levels. **Results:** The current study cleared out that treatment of rats with the *G. oblongata* extract prior to LPS injection significantly lowered serum cytokines, including NF-κB, MPO and LPO, and improved liver apoptosis through suppressing protein tyrosine kinase signaling pathway, and that may be due to antibacterial activity as well antioxidant capacity of *G. oblongata extract*. **Conclusion:** The present study was cleared out the possibility of administration of *G. oblongata red* algae as a *multi products source* for biotechnological, medical, nutraceutical and pharmaceutical applications due to highly antioxidant and anti-inflammatory capacities even although more investigations are required for separating, purifying and characterizing these bioactive compounds.

## Introduction

Inflammation is defined as a process that protects our body from foreign material and infection, such as fungi, virus and bacteria. Inflammation helps the body by producing white blood cells and other substances and resulting in expression of pro-inflammatory and suppression of anti-inflammatory genes [1]. The well-known symptoms of classic inflammation are pain, heat, redness, swelling and loss of function [2]. Studies recently showed that inflammation work through an advanced system and has a wide effect on different physiological process and human pathology. Although inflammatory response plays a pivotal role in protecting cellular physiological conditions, it is essential that inflammation is tailored to the initiating stress and resolves in a timely and controlled way, to avoid pathology associated with chronicity. If the inflammatory response does not shut down in a timely way, excessive inflammatory conditions can lead to various health problems [3].

Marine algae are important and valuable sources of structurally diverse biomaterials having antioxidant, anti-inflammatory, anticoagulant and antithrombotic activities among others [4–6]. Because free radicals resulted from oxidative stress have important effect in inflammatory reactions process and also in carcinogenesis, the marine algal natural bioactive compounds have been representing potential for use in different area and applications such as anticancer, antioxidant and anti-inflammatory drugs candidates. The bioactivities of marine algae have been evaluated both *in vitro a*nd *in vivo* and revealed numerous health-promoting effects, including antioxidative, anti-inflammatory, antimicrobial and anticancer effects (Rea et al., 2018). Additionally, marine algae contain large amounts of complex carbohydrates that can be considered as prebiotics [7]. Moreover, marine algae are rich in dietary fiber, minerals, lipids, proteins, omega-3 fatty acids, essential amino acids, polysaccharides, and vitamins A, B, C and E. Sulfated polysaccharides (PLS) are identified as highly beneficial biologically from studies demonstrating their antioxidant and anti-inflammatory effects [8].

The Red Sea is considering as the richest and most potentially productive source of marine environments. Marine organisms that live in the Red Sea are able to tolerate extreme changes in temperature, salinity, moisture and wave action to survive [9–16]. Marine organisms are able to survive in these extreme conditions and they produce a wide variety of primary and secondary metabolites which cannot be found in other organisms [17]. Marine bioactive natural products compounds can be derived from a variety marine sources, for examples marine macro- and microalgae, plants, seaweeds and sponges, all of which consist of various array of their own powerful and unique biomolecules [18–28]. Red algae are environmentally considerable as primary providers and producer of the structural habitats for many other marine organisms, and their important role is in the primary incorporations and protecting, maintenance and keeping of coral reefs. The presence of different bioactive molecules in seaweeds is expected due to environmental conditions and the inhabitation of these marine organisms in natural aquatic communities, where there are inhibitory competitions happen between both producers and consumers lead to release chemical weapons with highly target and powerful effect when diluted with many folds with water [29]. As a matter of fact, they produce a broad variety of unique potent substances, which include among others, carotenoids, terpenoids, xanthophylls, chlorophylls, phycobilins, polyunsaturated fatty acids, polysaccharides, vitamins etc. exhibiting potential beneficial use and stable properties for therapeutic use with low potential toxicity including antioxidant, anti-inflammatory, antiallergenic, antiaging and antiwrinkling effects and ultraviolet protection [5,6,25–28]. Moreover, the high level of minerals, vitamins, essential amino acids, indigestible carbohydrates and polyphenols, as well as their bioactive metabolites in the red algae and their relatively easy cultivation makes them attractive [30].

The objective of the present study was to investigate the red algae natural products by using GC-MS and their potential for prevention LPS-induced liver and kidney inflammation and injuries.

## Materials and methods

### Area of study area

**Figure 1:**
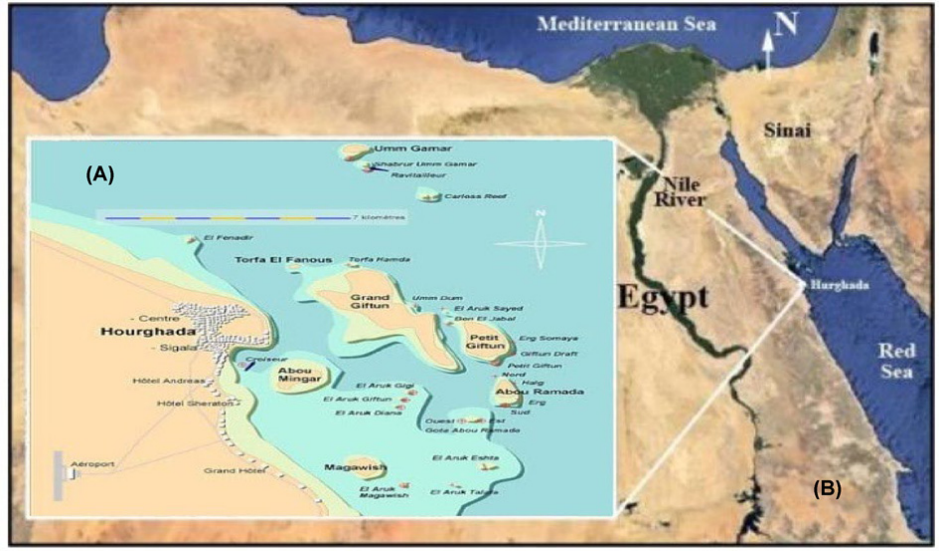
Location of Hurghada on the Egyptian Red Sea coast (A), and the sampling area at Hurghada (B)

The Egyptian coast of the Red Sea extends from 22° N near the Egyptian borders extending from Suez at the entrance of the Gulf of Suez passes from north Hurghada south to the Sudanese border and includes a number of offshore islands and west into the Gulf of Aqaba. The main threats to the Red Sea marine environment are land-based activities, which including coastal development and urbanization [31–38]. It is a major international shipping lane linking eastern and southern Asia with the Middle East and Europe. It is one of the warmest water bodies on earth creating a favorable condition for the growth of several marine ecosystems (Figure 1A). Hurghada is placed on the Red Sea south of Suez at about 350 km. It spread for about 36 km. The resort is a destination for more than 2.5 million visitors each year and brings more than 3 billion dollars to Egypt’s economy annually (Figure 1B). Despite the high biodiversity of the Red Sea, especially within the Egyptian borders, still, the research on the Red Sea biologically active compounds from different marine organisms is very scarce and underestimated in terms of the wealth of its natural characteristics [18–20,22–28].

### Equipment’s and chemicals

GC–MS (Thermo, U.S.A.), Mlutimode reader, and IR were applied. Different chemicals and solvents such as potassium ferricyanide, ferric chloride, sodium hydroxide, chloroform, glacial acetic acid, ferric chloride solution, sulphuric acid, folin–Ciocalteau, vanillin, methanol, hydrochloric acid, *n*-hexane, hydrogen peroxide, nitric acid, iron, zinc, cobalt, manganese and selenium etc. β-carotene, catechin, (+)-quercetin, sodium nitrite, aluminum chloride and gallic acid were purchased from Sigma Aldrich.

### Sample collection and extraction of bioactive secondary metabolites

*G. oblongata* red algae samples were collected from Hurghada-Sheraton at Latitude of 27 11 37.5 and Longitude of 33 50 48.4, during spring, 2019 (Figure 1B). The sample was translocated to the laboratory directly in sterile polyethylene bags. The identification of the seaweeds has been done by a NIOF team from the Hydrobiology lab at the Marine Environment Division. The fresh collected *G. oblongata* samples were washed with distilled water to remove extraneous materials, air dried for 10 days and occasionally turning them to avoid fungal growth. The *G. oblongata* samples were grind using IKA WEREKE M20 milling machine. The dried powdered *G. oblongata* (200 g) were soaked in 500 ml of hydride ethyl acetate in a 100 ml blue cape bottle. The content was vigorously shaken after that left in sonication bath for approximately 30 min and allowed to stand for 24 h. The mixture was filtered using a clean handkerchief cloth and re-filtered using Whatman No. 42 (125 mm) filter paper. The extraction was rotary evaporator and produced extract was then lyophilized.

### Identification and phytochemical screening of *G. oblongata* marine extract using gas chromatography-mass spectrometry (GC-MS) analysis

The *G. oblongata* marine extract was analyzed by GC-ITQ-MS using a Thermo Trace GC Ultra TM gas chromatograph system (Thermo Scientific, U.S.A.) according to the methods described by Nabil-Adam et al. [6]. The identification and interpretation of *G. oblongata* marine extract results of mass-spectrum GC-MS were done by comparing their mass spectra with those obtained from the NIST (National Institute of Standards and Technology) mass spectral database as conducted using the database of National Institute Standard and Technology (NIST).

### 2,2′-azino-bis (3-ethylbenzothiazoline-6-sulphonic acid) (ABTS^+^) radical scavenging activity assay of *G. oblongata* marine extract

The ABTS^+^ free radical decolonization assay developed according to Chakraborty and Paulraj et al. [40].

### Experimental animals, experimental design and tissue preparation

Forty (40) BALB/C mice weighing between 30 and 40 g were obtained from the animal house of the Institute of Theodor bilharz, Egypt. The protocol conforms to the guidelines of the National Institutes of Health (NIH). They were kept in plastic cages, each cage containing five animals. The animals were maintained in plastic cages, fed with standard laboratory chow obtained from the same unit, and water given ad libitum. The experimental method described by Abd El Moneam et al. [20]. The animals were randomly divided into four groups of 10 rats each and every group were placed in two cages. Group A: They received saline solution intraperitoneal (i.p) for one week and served as negative (–ve) control group. Group B: They received i.p 5 mg/kg, of LPS and served as induction group (+ve) control group. Group C: They received i.p 200 mg/kg, of *G. oblongata* algal extract for 2 h before LPS treatment; LPS+ algal extract and served as protected group. Group D: They received i.p 200 mg/kg, of *G. obligate* extract and served as positive (+ve) control group. At the end of the experiment, rats were anesthetized using diethyl ether. At the end of each week of treatment, blood samples were collected from mice in each group according to Abdel-Moniem et al. [21,22]. The liver and kidney tissues were weighed and cleaned from blood as well as adhering matters by washing in cold isotonic saline where 1 g of liver tissue was homogenized in 5 volumes of cold 0.1 M sodium phosphate buffer saline (PBS), pH 7.4 using mortar at 4°C. And the preparation was done according to Abdel-Moniem et al*.* [21,22]. The crude liver homogenate for the determinations of malondialdehyde (MDA), total antioxidant capacity, MPO, PTK. The supernatant was subdivided into three portions and stored at −20°C until used for subsequent determinations

## Biochemical measurements

### Determination of liver function

The AST and AST were determined according to the method of Reitman and Frankel [41]. Albumin level was determined according to the method of Doumas et al. [42]. Total bilirubin was determined according to the method of Walter and Gerad [42]. Total proteins were determined by means of the Biuret reaction as described by Gornall et al. [44].

### Biochemical measurements and assessment of kidney function tests

Serum creatinine was determined using Henry et al. [45] method. Serum urea concentration was determined enzymatically by the modified Berthelot reaction as described by Patton and crouch [46].

### Antioxidant activity and oxidative stress biomarker

#### Determination of total antioxidant capacity (TAC) and lipid per oxidation (LPO)

Determination of total antioxidant capacity was determined spectrophotometrically at 510 nm according to Koracevic et al. [47], where the lipid per oxidation (LPO) was determined according to Draper and Hadley [48].

#### Assessment of inflammatory and cancer biomarker

The tyrosine kinase activity was determined using PTK KIT from TAKARA.NF-κB was assayed according to the method described in commercial kit (Invitrogen, Camarillo). The absorbance was measured at 450 nm against blank using an ELISA reader (RayBiotech, Canda). The MPO was determined according to Peroxidase activity with 3, 3′, 5, 5′-Tetramethylbenzidine (TMB, Sigma) was measured according to Pulli et al. [49].

#### Histopathological study

The liver and kidney tissues were fixed in formalin and dehydrated in ascending grades of alcohol, then immersing the tissues in xylene for 1 h three times and impregnation in melted paraffin, and after that putted in oven at 60°C for approximately 1 h. The samples were left to solidify at RT after embedded in paraffin. Sections of 5 μm thick were cut and mounted on clean glass slides using microtones. The liver and kidney sections were prepared then stained with hematoxylin and eosin (H&E), and the alterations in histology were investigated according to Griffith and Farris [50].

#### Ethical for animal experimentation

The Ethical Animal treatment according to the guideline of ethical animal treatment in National Institute of Health (NIH) was followed in adherence to established protocols and all animal protocols were approved and accomplished by the Institutional Animal Care and Use Committee (IACUC) in Alexandria University (ethical approval reference number: AU- 0304926).

### Statistical analysis

The data were given as individual values and as means (X) ± standard deviation (SD) for seven animals in each group. Comparisons between the means of various treatment groups were analyzed using least significant difference (LSD) test. Differences were considered significant at *P*≤0.05. All statistical analyses were performed using the statistical software SPSS and prism .

## Results

**Figure 2:**
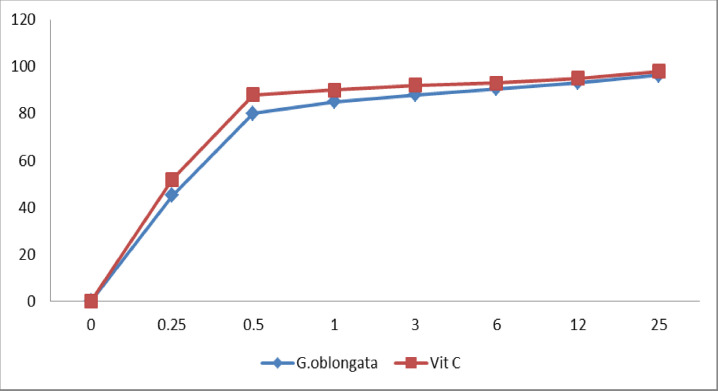
The total antioxidant capacity of *G. oblongata* using ABTS model compared with vitamin C

The phytochemical screening of *G. oblongata marine* extract using GC-MS showed a great diversity of different bioactive compounds such as carotenoids, alkaloids, vitamins, fatty acids and hormones (Table 1 and Figure 2).

**Table 1:** The different identified bioactive compounds by using GC-MS screening of *G. oblongata* marine extract

Compound Name	RT	Prob.	Area	Area %	Compounds nature	The reported biological activity
2-Myristynoyl pantetheine	1.33	15.23	848715.00	0.24	Vitamins	Antioxidant and anti-inflammatory [78]
Oleic Acid	1.64	5.37	78500.64	0.02	Fatty acids	Anti-inflammatory [79]
Heptanoic acid, docosyl ester	3.37	3.52	283357.70	0.08	Fatty acids	Antioxidant anticancer and anti-inflammatory [80]
5,8,11,14-Eicosatetraynoic acid	3.45	5.00	1055770.75	0.30	Fatty acids	Antioxidant anticancer and anti-inflammatory [80]
Dodecane, 5,8-diethyl-	3.65	10.03	39172.25	0.01	Fatty acids	Antioxidant anticancer and anti-inflammatory [80]
Oleic acid, 3-(octadecyloxy) propyl ester	3.71	6.67	182597.13	0.05	Monounsaturated omega-9 Fatty acids	Antioxidant anticancer and anti-inflammatory [80]
Ethyl iso-allocholate	4.27	6.26	371088.37	0.10	Steroids	Antioxidant and anti-inflammatory [81]
Lochneridine	5.65	11.80	129134.93	0.04	Alkaloids	Anti-inflammatory and antioxidants [81]
Lycopene	5.65	9.51	129134.93	0.04	Carotenoids and natural dyes	Anti-inflammatory, antioxidant and anticancer [68]
N-2,4-Dnp-L-arginine	6.90	11.73	730119.87	0.21	Amino acids	Antioxidant anticancer and anti-inflammatory [80]
Akuammilan-17-oic acid, methyl ester	6.90	4.63	730119.87	0.21	Fatty acids	Antioxidant anticancer and anti-inflammatory [80]
Benzoyl-L-arginine amide	7.00	6.52	336407.26	0.09	Amino acids	Antioxidant anticancer and anti-inflammatory [80]
Dodecane, 5,8-diethyl-	7.38	8.24	207625.46	0.06	Fatty acids	Antioxidant anticancer and anti-inflammatory [80]
Dodecanoic acid, tetradecyl ester	7.38	3.75	207625.46	0.06	Fatty acids	Antioxidant anticancer and anti-inflammatory [80]
Octadecane, 3-ethyl-5-(2-ethylbutyl)-	7.91	7.71	1400102.23	0.39	Fatty acids	Antioxidant anticancer and anti-inflammatory [80]
Heptadecane, 9-hexyl-	8.01	23.53	450147.45	0.13	Fatty acids	Antioxidant anticancer and anti-inflammatory [80]
Glucobrassicin	8.26	8.67	97248.99	0.03	glucoinalted	glucosinolates can stimulate the body's own natural antioxidant systems, technically called Phase II enzymes. As such, glucosinolates act as indirect antioxidants triggering the liver to produce detoxifying enzymes that block free-radical attack on DNA. Also severy studies indicate their activity as anti-inflammatory
Aldosterone	8.31	8.60	194877.76	0.05	Hormones	Coproducing Adrenal Adenoma in Primary Aldosteronism [82]
Hexadecanoic acid, 1-(hydroxymethyl)-1,2-ethanediyl ester	9.30	11.46	684566.51	0.19	Fatty acids	Antioxidant anticancer and anti-inflammatory [80]
à-D-Xylofuranose, cyclic 1,2:3,5-bis(butylboronate)	10.45	15.55	820042.46	0.23	Carbohydrates	Anti-inflammatory, antioxidant and anticancer [68]
Ethyl 9-hexadecenoate	10.95	5.90	3196905.07	0.90	Fatty acids	Antioxidant anticancer and anti-inflammatory [80]
Androst-5,7-dien-3-ol-17-one	17.91	6.31	217424.95	0.06	Hormones	Antioxidants [80]
Eicosanebioic acid, dimethyl ester	18.32	7.24	490162.97	0.14	Fatty acids	Antioxidant anticancer and anti-inflammatory [80]
Phorbol	19.73	20.24	349250.47	0.10	Diterpene	Anti-inflammatory, antioxidant and anticancer
DL-Cystine	20.09	6.86	55387.63	0.02	Amino acids	Antioxidant anticancer and anti-inflammatory [80]
5,8,11,14-Eicosatetraynoic acid	27.25	8.97	531005.71	0.15	Fatty acids	Antioxidant anticancer and anti-inflammatory [80]
Xanthumin	27.59	5.67	244505.99	0.08	Carotenoids and natural dyes	Anti-inflammatory, antioxidant and anticancer [68]
Astaxanthin	28.51	10.05		0.05	Carotenoids and natural dyes	Anti-inflammatory, antioxidant and anticancer [68]
Benzoic acid, 4-methyl-, [4-(methoxycarbonyl)phenyl]methyl ester	33.78	7.69		0.26	Phenolic derivatives	Anti-inflammatory, antioxidant and anticancer
11-Heneicosanone	35.09	6.26		0.37	Fatty acids	Antioxidant anticancer and anti-inflammatory [80]
á-Hydroxyquebrachamine	35.23	10.34	332563.89	0.09	Alkaloids	Anti-inflammatory, antioxidant and anticancer
l-Glutamic acid, monobenzyl ester	35.28	10.94		0.07	Amino acids	Antioxidant anticancer and anti-inflammatory [80]
Cholestan-3-one, 4,4-dimethyl-, cyclic 1,2-ethanediyl acetal, (5à)-	35.53	16.59	422479.71	0.12	saturated tetra cyclictriterpene	Antioxidant anticancer and anti-inflammatory [80]
Canthaxanthin	36.25	5.26		0.17	Carotenoids	Anti-inflammatory, antioxidant and anticancer [68]
1,4,7-Androstatrien-3,17-dione	36.50	5.58		0.02	Hormones	Anti-inflammatory, antioxidant [68]
Folic Acid	36.90	9.88		0.16	Vitamins	Antioxidant and anti-inflammatory [78]
Stearic acid, 3-(octadecyloxy)propyl ester	38.48	6.26		0.17	Amino acids	Antioxidants and anti-inflammatory [83]
Ethyl iso-allocholate	1.52	5.48	116195.34	0.03	Steroids	Antioxidants and anti-inflammatory [83]
Spiculesporic acid	0.33	7.58	29624.78	0.01	γ-butenolide	Antioxidants and antimicrobial [84]
Retinoic acid, methyl ester	0.82	5.39	1621522	0.44	Vitamins	Antioxidant and anti-inflammatory [78]
5,8,11,14-Eicosatetraynoic acid	3.98	5.82	83212.1	0.02	Fatty acids	Antioxidant anticancer and anti-inflammatory [80]
.psi.,.psi.-Carotene, 1,1′,2,2′-tetrahydro-1,1′-dimethoxy-	6.36	10.52	45278.33	0.01	Carotenoids and natural dyes	Antioxidant anticancer and anti-inflammatory [68]
Oleic Acid	11.1	5.25	1235479.1	0.33	Fatty acids	Antioxidant anticancer and anti-inflammatory [80]
Glucobrassicin	13.77	7.55	84398.25	0.02	glucosinolate	Antioxidants, anti-inflammatory [85]
5,8,11,14-Eicosatetraynoic acid	11.96	17.64	554245.64	0.15	Fatty acids	Antioxidant anticancer and anti-inflammatory [80]
Gibberellic acid	11.96	10.69	554245.64	0.15	Diterpene	Anti-inflammatory, antioxidant and anticancer [80]

### The total antioxidants capacity using ABTS model compared with vitamin C

The total antioxidant capacities against ABTS using different concentrations of the *G. oblongata* were summarized in Figure 2. The results of the present study cleared out that the *G. oblongata* marine extract exhibited a high antioxidant capacity at 0.5 mg of 88% inhibition activity comparing with VitC that was showed 88% inhibition activity at the same concentration.

### Biochemical measurements and assessment of liver and kidney functions

#### Liver functions

**Figure 3:**
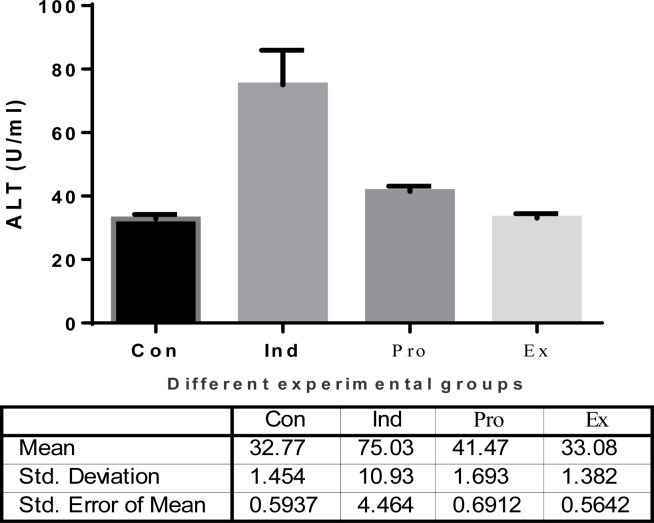
The effect of *G. oblongata* extract on the serum ALT levels (U/ml) in the different mice groups

**Figure 4:**
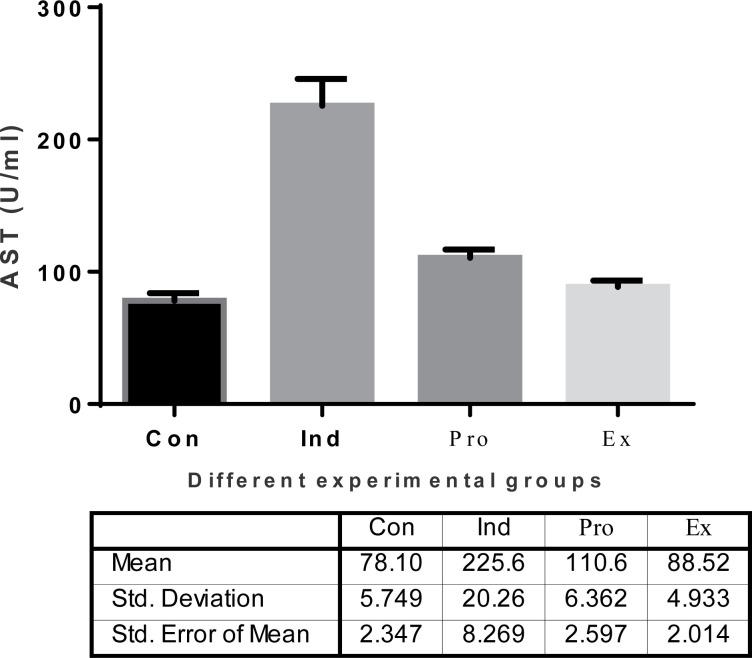
The effect of *G. oblongata* extract on the serum AST levels (U/ml) in the different mice groups

The results of the current study showed that LPS-treated group (induction group) had a highly significant (*P* < 0.01) increase in both the serum ALT and AST activities (75.3.22  ±  20.26 U/ml and 225.6  ± 10.39 U/ml, respectively) compared with the -ve control group (32.77 ± 1.454 U/ml and 78.1 ± 5.74 U/ml, respectively). On contrast, *G. oblongata* marine extract treatment group (extract group) did not show a significant change in the activities of ALT and AST compared with the control group. Furthermore, the present study results revealed a highly significant (*P* < 0.01) decrease in the activity of serum ALT and AST in the protected group (*G. oblongata* + LPS) group III (41.47.20 ± 1.69 U/ml and110.6 ± 3.62 U/ml) respectively compared to the induction group (LPS-treated) (75.03±10.93 U/ml and 225.6 ± 10.39 U/ml, respectively) (Figures 3 and 4).

**Figure 5:**
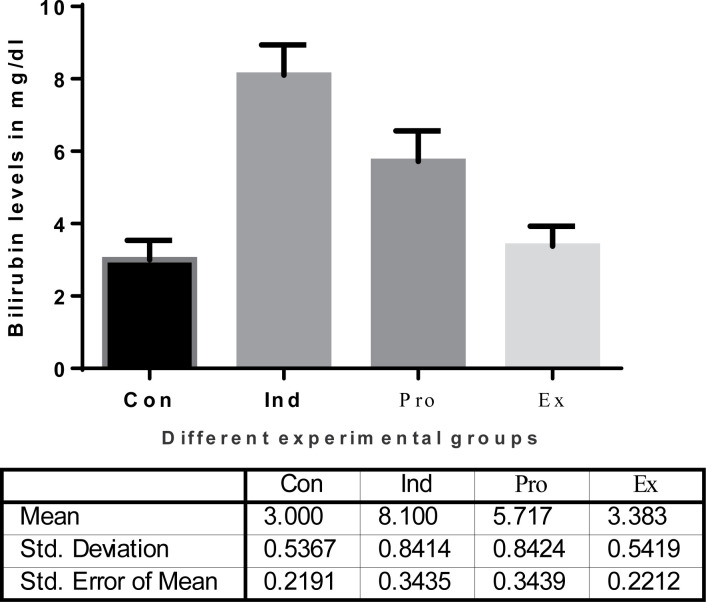
The effect of *G. oblongata extract* on the serum bilirubin levels (mg/dl) in the different mice groups

**Figure 6:**
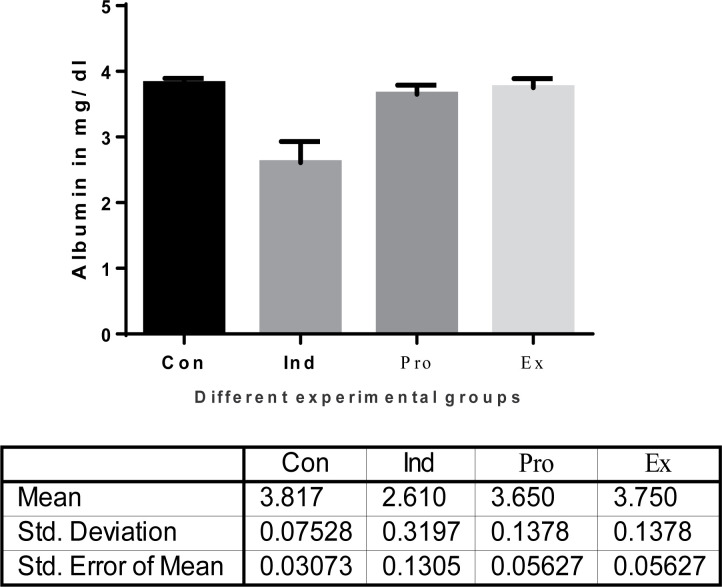
The effect of *G. oblongata* extract on the total albumin levels (mg/dl) in the different mice groups

**Figure 7:**
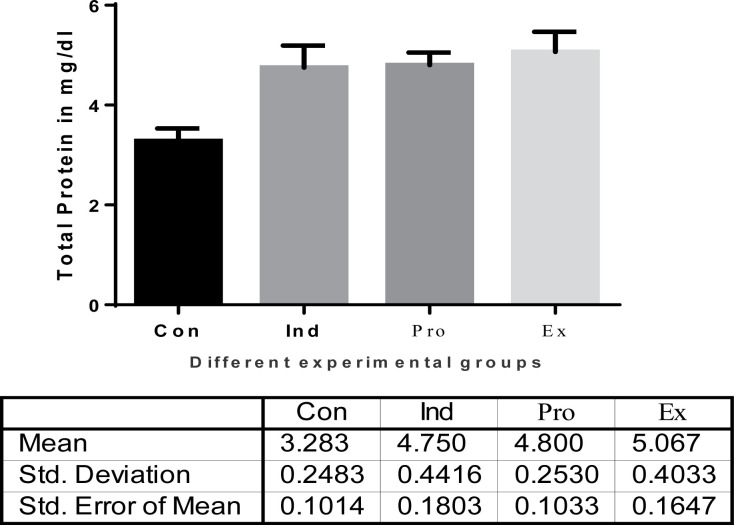
The effect of *G. oblongata* extract on the total protein levels (mg/dl) in the different mice groups

Moreover, there was a significant decrease in serum bilirubin activity in the protected group (*G. oblongata* + LPS) group (5.717 ± 8. mg/dl) compared with the induction group (LPS-treated) (8.1 ± 0.8414 mg/dl) (Figure 5). The current results indicated also a highly significant (*P* < 0.01) increase (4.75 ± 0.4416/dl) in the hepatic total proteins of the induction group (LPS-treated) mice (Figure 6). Additionally, the total albumin was significantly decreased in the induction group (2.6 ±0.3197). On contrast, there was a non-significant increase in the liver total albumin in mice pretreated with *G. oblongata* extract (3.65 ± 1.378 g/dl) compared with that of the control (3.187± 0.07528 mg/dl) group (Figure 7). Meanwhile, there was a highly significant increase in the hepatic total proteins as well as albumin in the protected group (*G. oblongata* + LPS) group compared with the LPS-treated (induction group).

#### Kidney functions

**Figure 8:**
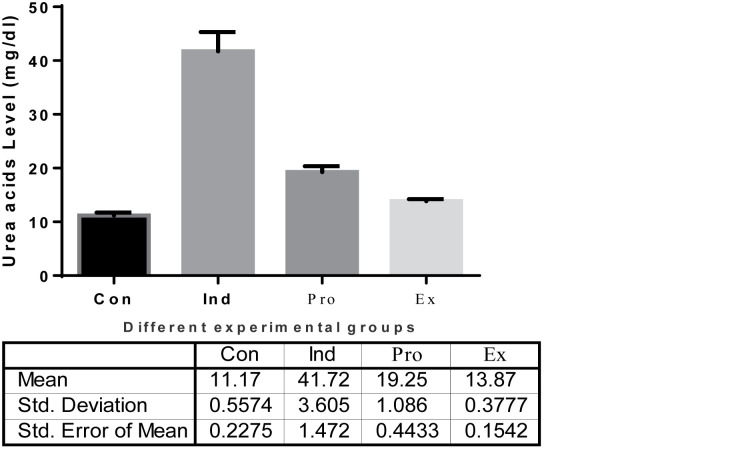
The effect of *G. oblongata* extract on the urea levels (mg/dl) in the different mice groups

**Figure 9:**
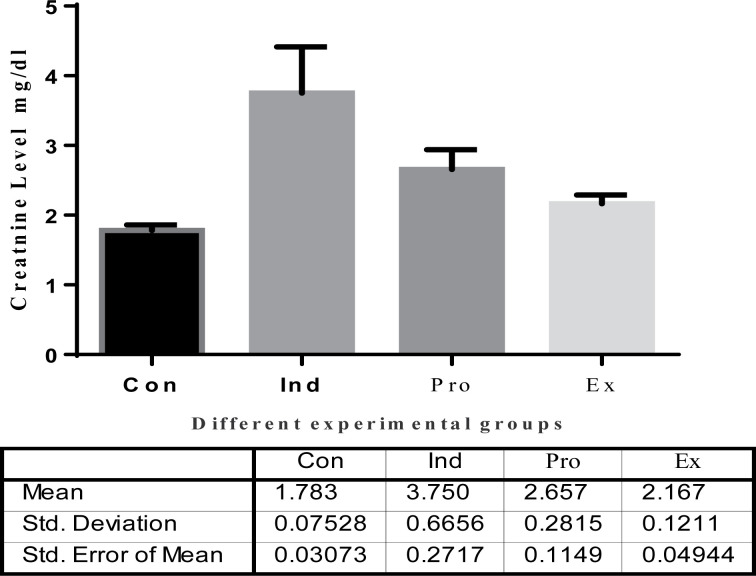
The effect of *G. oblongata* extract on the creatinine level (mg/dl) in the different mice groups

**Figure 10:**
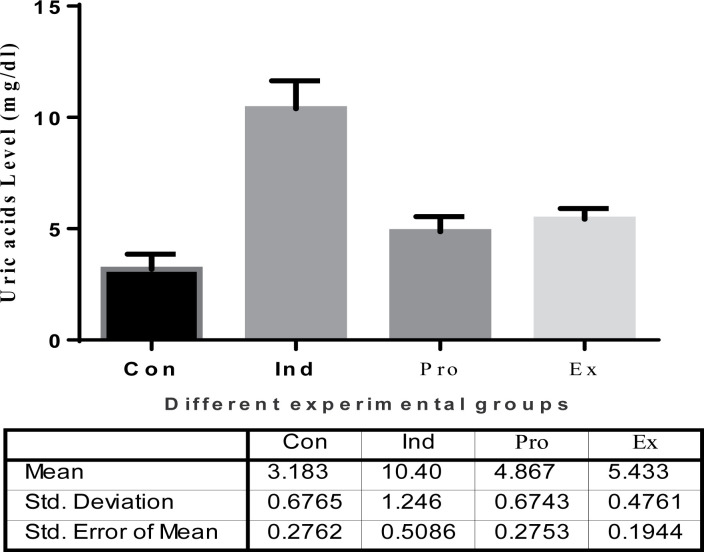
The effect of *G. oblongata* extract on the total uric acid (mg/dl) in the different mice groups

The results of kidney functions are represented in Figures 8–10. The results of urea showed a highly significant (*P* < 0.01) increase in the serum urea (41.72  ± 3.605 mg/dl) and creatinine (3.750 ± 0.66 mg/dl) in the LPS-treated mice compared with the control levels (Figures 8 and 9), whereas treatment of mice with the *G. oblongata* extract prior to LPS injection significantly diminished serum urea (19.25 ± 1.086 mg/dl) and creatinine (2.657 ± 0.281mg/dl) compared with LPS-treated group. Additionally, the uric acids were significantly increased in the induction group (10.40 ± 1.246 mg/dl) compared with the control group (3.183 ± 0.6765 mg/dl). On contrast to the protection group, the uric acid was significantly decreased compared to the induction group (Figure 10).

#### Oxidative and antioxidant status, malondialdehyde (MDA) and total antioxidant capacity (TAC)

**Figure 11:**
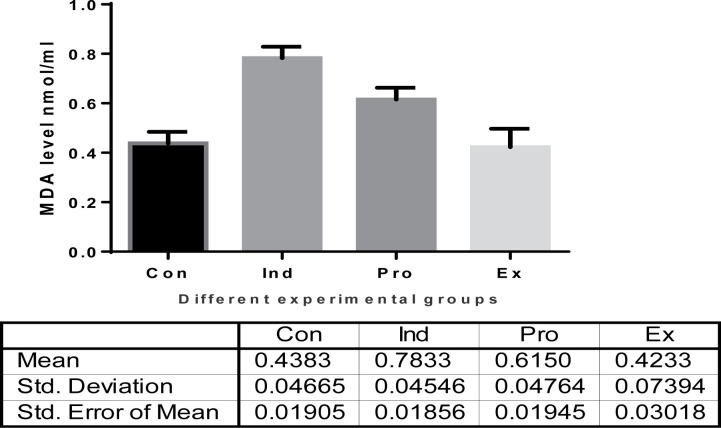
Malondialdehyde (MDA) levels (nmol/m) in the different mice groups

**Figure 12:**
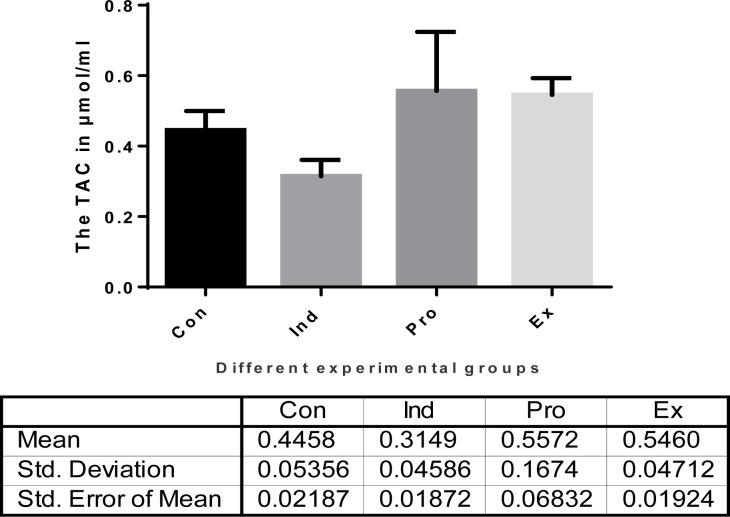
Total antioxidant capacity (TAC) levels (µmol/ml) in the different mice groups

Injection of mice with LPS caused a highly significant (*P* <0.01) increase in the MDA level (0.7833 ± 0.45 nmol/ml), while the mice that received *G. oblongata* marine extract had a non-significant increases in the MDA level (0.4233 ± 0.739 nmol/ml) compared to the control ones (0.438 ± 0.466 nmol/ml). Furthermore, there was a highly significant (*P*<0.01) inhibition of MDA production of the combination group (*G. oblongata* + LPS) (0.615 ± 0.0476 nmol/ml) compared with the induction group (LPS-treated) (Figure 11). The TAC level revealed a highly significant (*P* <0.025) decrease (0.31 ± 0.05 µmol/ml tissue) in the induction group (LPS-treated) compared with those of the control ones (0.45 ± 0.05 µmol/ml). Pretreatment of mice with *G. oblongata marine* extract prior to LPS injection led to a highly significant (*P*<0.001) increase in TAC level (0.56 ± 0.17 µmol/ml) compared with the induction group (LPS-treated group) (Figure 12).

#### Impact of *G. oblongata* marine extract on the apoptosis biomarkers

**Figure 13:**
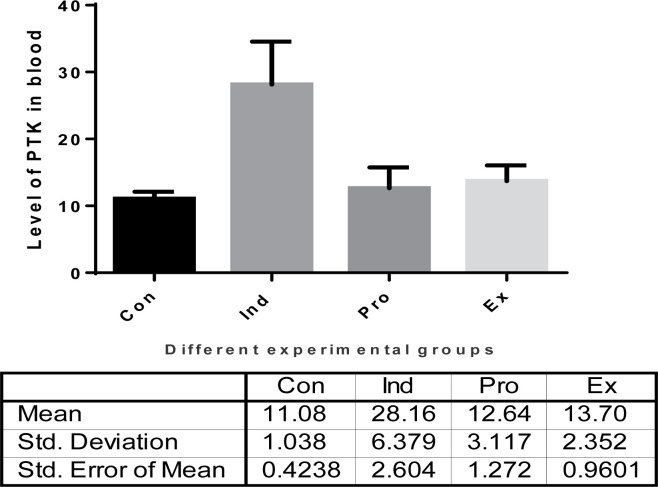
The effect of *G. oblongata* marine extract on the protein tyrosine kinase (PTK) levels

The effect of *G. oblongata* marine extract on the hepatic protein tyrosine kinase (PTK) level of induced toxicity group (group II) is illustrated in Figure 13. The PTK levels were found to be significantly increase in group (II) (LPS treated group) mice by 59.82% compared with group I (11.08 ± 1.04 vs. 27.58 ± 3.83, *P*<0.001). Administration of *G. oblongata* marine extract in group III showed a significant reduction in PTK by 55.11% compared with group II (induction group) (28.16 ± 6.38 vs. 112.64 ± 3.12, *P*<0.001). In addition, the administration of *G. oblongata* marine extract alone in group IV (+ve control group), a non-significant increase in the PTK level from (11.08 ±1.04) to (13.7 ± 2.35). The levels of PTK activities in groups (I), (III), and (IV) showed a non-significant increase and/or decreased when compared with each other.

#### Impact of G. oblongata on the inflammatory biomarkers

##### The transcription nuclear factor-kappa (NF-κB) biomarker

**Figure 14:**
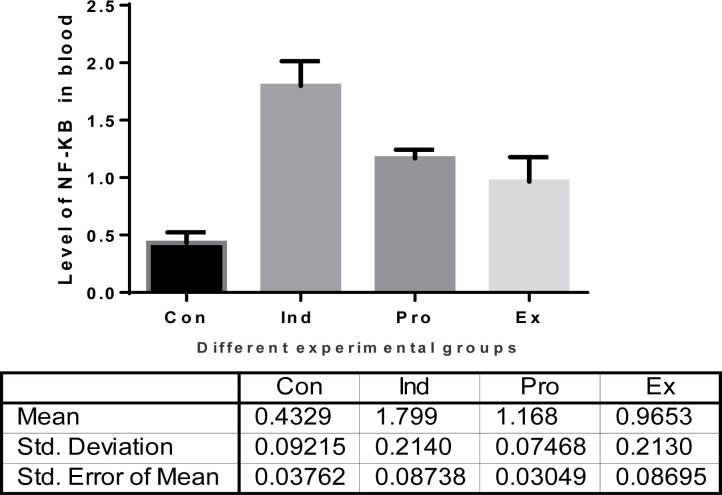
The effect of *G. Oblongata* marine extract on the NF-κB biomarkers levels

The effect of algal *G. oblongata* marine extract on the activities of NF-κB in mice serum of LPS treated group (II) compared with the other experimental groups (I, III, IV) is presented in Figure 14. In group (II) the activity of NF-κB (1.80 ± 0.21) was found to be significantly increase than that of the –ve control group (group I) mice by 76.1% (0.43 ± 0.09). On contrast, the algal *G. oblongata* marine extract pretreatment group (group IV) at 200 mg/100 b.w/day for 7 days had a significant lower NF-κB by 35% (1.17 ± 0.07) compared with group (II). Furthermore, the activities level of NF-κB in group (III) showed a non-significant increase or/and a decrease when compared to their corresponding values either of group (I) or group (IV) (+ve control group) (Figure 14).

##### Myeloperoxidase (MPO) biomarker

**Figure 15:**
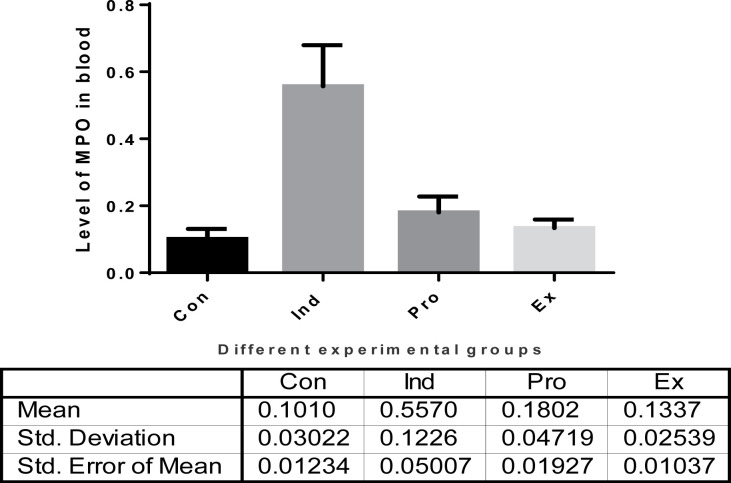
The effect of *G. oblongata* marine extract on the MPO biomarkers levels

**Figure 16:**
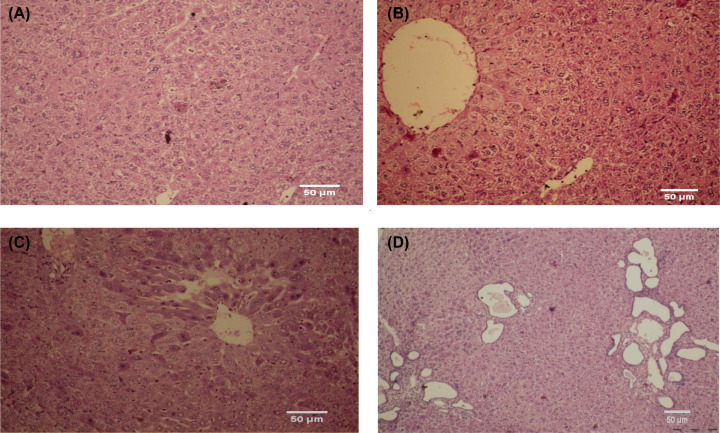
The effect of *G. oblongata* marine extract in the histopathology of liver tissues different experimental groups The Figure 16 showing the difference and comparison between studies groups comparing with control (**A**) where in the (**B**) in induction group showing abnormal histopathological appearance with intense heavy portal lymphoplasmacytic, inflammatory infiltrate changes of hepatocytes, and that in contrast with (**C**) (protection group) which showing great improvement of damage signs.by decreasing the inflammation and portal infiltration greatly also the enlargement in portal vein was reduced significantly.addationally the extract group showing normal architecture with no sign of abnormality.Addationally figure (**D**) showed no abnormal appearance in extract group.

The effect of *G. oblongata* marine extract on the activities of MPO in mice serum of LPS-treated group (II) compared with the other experimental groups (I, III, IV) is given in Figure 15. In group (II) the activity of MPO (0.56 ± 0.12) was found to be significantly increase than that of the –ve control group (group I) mice by 76.1%, (0.10 ± 0.03). On contrast the *G. oblongata* marine extract pretreatment (group IV) at 200 mg/100 b.w/day had a significant lower MPO by 67.8% (0.18 ± 0.05) when compared with group (II). Furthermore, the activities level of MPO in group (III) showed a non-significant increase when compared with their corresponding values either of group (I) or group (IV) (+ve control group) (Figure 16).

##### The histopathology of liver tissue in different experimental groups

The present study showed that the liver tissues of the control group had a normal hepatic architecture formed of cords of hepatocytes separated by hepatic sinusoids (H&E 400×). On the meantime, the extract group had also preserved hepatic architecture (H&E 400×). On contrast, the induction group of liver sections showed abnormal histopathological appearance. The livers of rats treated with LPS demonstrated many histopathological features represented by intense heavy portal lymphoplasmacytic, inflammatory infiltrate changes of hepatocytes. (H&E 400×) as well as the observed hydropic alteration in hepatocytes, and portal lymph plasmatic infiltrate and parenchymal hydropic alterations with apoptosis and binucleated cells. The protection group showed a great improvement (Figure 16).

##### The histopathology of kidney tissue in different experimental groups

**Figure 17:**
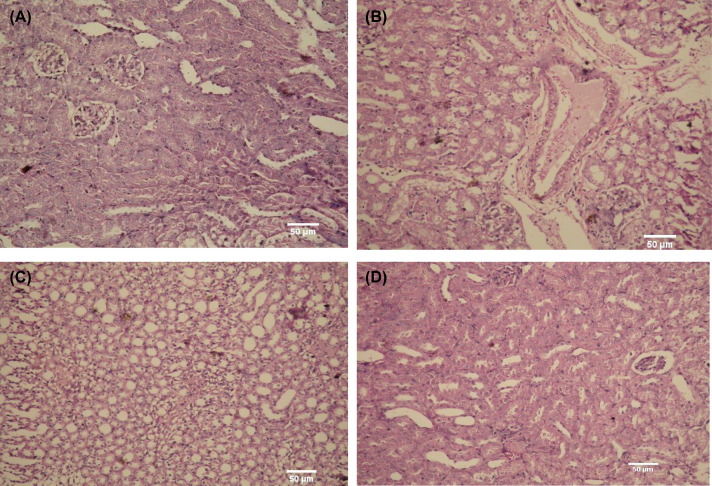
The effect of *G. oblongata* marine extract in the histopathology of kidney tissues different experimental groups The (**B**) showing different damage stages histological evaluation in the LPS group showed clear features of tubular injury, with evidenced of tubular necrosis, additionally there are loss of the brush border as well as tubular dilation in the cortex and outer medulla in contrast with protection group on (**C**) which showing great improving in inflammation and tubular necrosis additionally the extract group in (**D**) showing normal architecture as in control group in (**A**).

The present study showed that the kidney tissues of the control group had normal histological features as glomerulus of renal corpuscle, distal convoluted tubules, renal corpuscle, renal tubules, glomerulus of renal corpuscle with its filtration space, proximal convoluted tubules, and distal convoluted tubules all in normal and intact. Furthermore, the extract group had the same pattern as the control group. On contrast, the induction group exhibited severe distortion of many renal corpuscles. The cells of proximal and distal convoluted tubules had also cytoplasmic vacuoles and many nuclei appear pyknotic. Moreover, the presence of a atrophy for others with obliteration of the filtration spaces. The protection group showed a great improvement of infiltration and inflammation as the *G. oblongata* could reduce the number of proximal cells (Figure 17).

## Discussion

**Figure 18:**
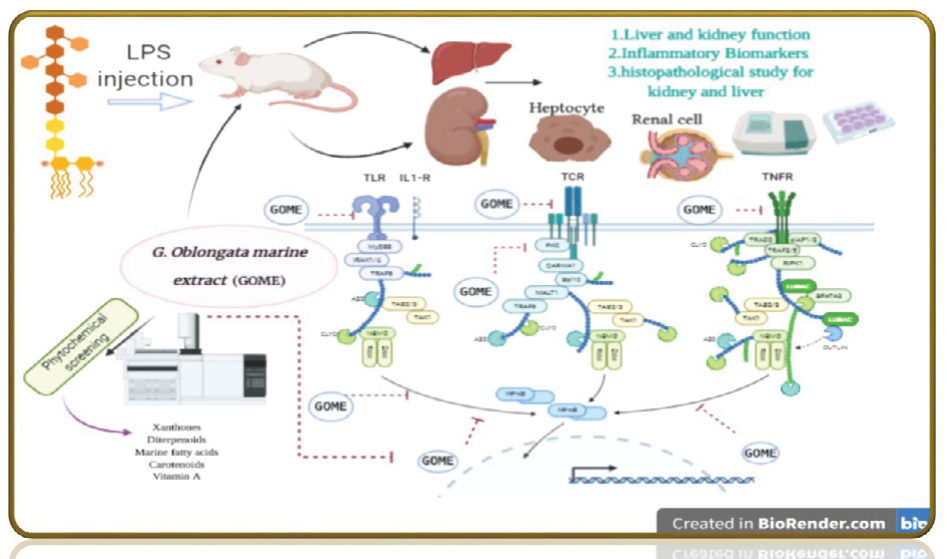
The schematic diagram showed the protective effect of ***G. oblongata*** against LPS-induced liver and kidney injuries through inhibition of different metabolic pathways (Created with BioRender.com)

Acute liver and kidney injuries are major problems nowadays as they consider as one of the most distributed medical problems in patients with critical state and lead to high mortality rates. Recent studies for both basic study research on pathophysiology and clinical studies have shown a complex association between the liver and kidney via the vascular microenvironment and related immune mediators [51]. The hepatorenal syndrome is one of many potential causes of acute kidney injury in patients with acute or chronic liver disease. The treatment with the *G. oblongata* marine extract on LPS-induced liver and kidney injury in mice revealed improvements in liver and kidney functions as proved in the current study (Figures 2–18). In addition to the anticancer activity and the total antioxidant capacity of the *G. oblongata* marine extract (Figures 2 and 3), the present study cleared out that the liver enzymes (transaminases) were elevated greatly in the induction group and that may be attributed to the effect of lipopolysaccharide (LPS) that stimulates the septic liver injury resulting in activation the transcription factor nuclear factor-kappa B (NF-κB) and leading to activation of many inflammatory genes, such as TNF-α and IL-1β [52,86]. NF-κB is one of the most important inflammatory cytokines, and know it is used as an indicator and biomarkers for inflammatory response in different diseases more over it become the target of many anti-inflammatory drug discovery programs. The administration of LPS in animal models induces the expression and release of NF-κB [52]. Most authors observed the NF-κB serum peak as early as 0.5–2 h after injection with LPS [8,4]. Previous studies showed that decreasing the flow of liver blood through portal shunt down regulates the systemic levels of NF-πB [52]. In rat model [52], the portal vein ligation decreased LPS-induced NF-κB [52]. These observations suggested that the severity of the LPS-response in terms of organ damage was observed as in in Figures 14,16 and 17. The NF-κB signal pathway lies at the center of the inflammatory and immune response [52]. In animal models, LPS administration induces the expression and release of NF-ÓB ROS to stimulate NF-ÓB signal through the IKK- classical dependent pathway and to activate inflammation as well as injury in various organs for examples of kidney and liver (Figure 18) [52]. We also observed the destructive effect of LPS oxidative stress and protective role of *G. oblongata* in the present study had an antioxidant as well as anti-inflammatory effect against the cytotoxicity induced by LPS. Level of expression of NF-κB and other inflammatory biomarkers in LPS-treated mice. The results revealed the *G. oblongata* had anti-inflammatory, liver and kidney protection effects through antioxidant effect of their content. In the present study, ALT, AST, bilirubin as well as total protein were highly elevated in the serum of LPS-induced mice (Figures 4-8). On contrast, total albumin was greatly decreased and that because LPS able to inhibit different cytochrome P450s as CYP2E1 at transcription and translation levels. In addition, CYP2E1 itself is also an effective enzyme for ROS production, exhibits enhanced NADPH oxidase activity, and elevated rates of production of O_2_^·−^ and hydrogen peroxide (H_2_O_2_) even in the absence of substrate (García-Suástegui et al. [53] and Ding et al. [54] reported that LPS-induced acute liver injury (ALI) via the Toll-like receptor 4 (TLR4) signaling pathway in the Raw264.7 cell line and in BalB/c mice as seen in schematic Figure 18.

LPS can inhibit several cytochrome P450s including CYP2E1 at transcription and translation levels. By producing increase amount of ROS, CYP2E1 sensitize the toxicity of hepatotoxins. LPS activates Kupffer cells to produce O_2_^·−^ via NADPH oxidase, which may synergize with CYP2E1‐mediated oxidative stress [86]. In agreement with the findings of Ito et al. [55] who showed that overproduction of ROS may cause lipid peroxidation and form MDA, the present study revealed a high increase in MDA production in induction group. LPS can also induce NO production; e.g., O_2_^·−^ derived from CYP2E1 can react with NO rapidly to form OONO. The present study results cleared out that the *G. oblongata* decreased OONO^−^ formation through lowering the production of O_2_^·−^ following inhibition of CYP2E1 activity. The present study showed that *G. oblongata* marine extract increased the survival rate of mice and attenuates the LPS-induced liver injury, which is indicated by pathology and serum liver enzymes. Moreover, the *G. oblongata* marine extract ameliorated hepatic oxidative stress indicators MDA and enhanced the level of total antioxidant capacity. Additionally, the *G. oblongata* marine extract also attenuated regional and systematic inflammation and further reduced apoptosis of hepatocytes. Mechanistic evidence was also investigated in the present study as the *G. oblongata* marine extract decreased MPO as well as MDA levels, anti-inflammatory/antioxidative pathway during oxidative stress, NF-κB pathway, and meanwhile, it suppressed PI3K/AKT signaling that reduced apoptosis [56]. Conclusively, the present study unveiled the protective role of the *G. oblongata* marine extract in LPS-stimulated oxidative reaction, inflammation, and apoptosis by suppressing NFkb/ROS as well as PI3K/AKT pathways through inhibition of PTK, suggesting its promising role in attenuating inflammation as well as the liver injury (septic endotoxemia) which is a process that levels of blood LPS are elevated. In the kidney results, the *G. oblongata* marine extract exerted its beneficial effect by reducing LPS effect which may be associated with inhibiting the Nfkb-mediated oxidative stress and programmed cell death in kidney cells. Apoptosis is an important pathological mechanism leading to kidney injury [57]. As expected, the LPS-induced kidney injury in the induction group represented by increasing apoptosis in the blood, and elevation in expression levels of PTK which is consistent with the effects of different secondary metabolites present in the *G. oblongata* marine extract possessing diverse biological effects on renal. These natural products from marine environment are present in different sources like plants, microorganisms, fungi, etc. Besides being non-toxic in nature they are considered to less expensive [18–28]. Seaweeds in general and red algae in particular have been reported for their potential role as potent anti-inflammatory and anticancer because of their rich and unique constituents [58–60], which agrees well with the present study findings (Table 1). The present study revealed the presence of different secondary metabolites such as marine Xanthones which exhibit wide spectrum of activities such as anticancer, anti-inflammatory, antidiabetic, etc. [61]. The phytochemical screening of *G. oblongata* marine extracts using GC-MS revealed the presence of diterpenoids including phytol and gibberellin (Table 1). Diterpenoids have been reported to have anti-inflammatory and antiproliferative properties [62]. In a previous study, Silva et al. [63–70] reported that phytol, which is a diterpene alcohol, could reduce neutrophil migration, cytokine levels, and oxidative stress in acute inflammation models and through inhibition of neutrophil migration is due to reduce IL‐1β and TNF‐α levels and oxidative stress. Gibberellic acid has also potent anti-inflammatory effect was mediated through reduced IκBα degradation and reduced NF-κB (p65) expression. Additionally, the Zinc-finger protein A20, induction by Gibberellic acid attenuates inflammation in airway epithelial cells, through its effect on NF-κB and IκBα [39–64]. Another class of marine secondary metabolites identified by GC-MS in the *G. oblongata* marine extract is the marine fatty acids (Table 1). The long chain fatty acids effect inflammation process via several of mechanisms; many of these mechanisms are intercede by associated with, changes in the fatty acid composition of cell membranes. Changes in the compositions of fatty acids can change membrane fluidity, alteration in gene expression due to change in cell signaling, also in the production of lipid. Many studies indicated that the cells participated in the inflammatory response are rich in the arachidonic acid *n*-6 fatty acid, but the compositions of arachidonic acid as well as of the *n*-3 fatty acids eicosapentaenoic acid (EPA) and docosahexaenoic acid (DHA) can be altered through oral administration of EPA and DHA which serve as the necessary substrates for the production of anti-inflammatory and inflammation resolving mediators (resolve, protections and treatments) whilst simultaneously inhibiting the transcription of pro-inflammatory genes. Modification and changing in the fatty acid composition of cells including in the inflammatory response also affects the production as well as synthesis of several peptide mediators of inflammation such as cytokines, adhesion molecules, etc. The fatty acid contents of involved cells in the inflammatory response effect, their function; the compositions of arachidonic acid, DHA and EPA showed to be specifically important. The anti-inflammatory effects of marine *n*-3 PUFAs suggest that they may be useful as therapeutic agents in disorders with an inflammatory component [65,66]. Carotenoids are the third class of marine secondary metabolites identified by GC-MS in the *G. oblongata* marine extract (Table 1). β-Carotene prevent or halt the oxidative stress, which has been reported to be the under-current mechanism for most of the modern epidemics like diabetes, hypertension, cardiovascular diseases, cancer, cataract and many other chronic diseases. They are proven to be antioxidants that not only scavenges various ROS, including singlet oxygen, the superoxide anion radical, the hydroxy radical, the peroxyl radical and nitric oxide which can attack other molecules to acquire electron and become stable, but also inhibit lipid peroxidation. It has been reported that the antioxidant as well as anti-inflammatory activity of β-carotene molecules, may be due to their ROS-scavenging activity and to elevation of the potency of the electrophile/antioxidant response element transcription system which may be due to their high number of conjugated dienes, which act as potent free radicals and ROS quenchers [67–69]. LPS is a strong inducer of oxidative stress production, and the excessive production of ROS is closely related to the apoptosis of kidney as well as liver cells leading also to release of different apoptotic factors, including cytochrome *C*, which in sequence lead to permeability damage of the mitochondrial membrane. In the present study, mice injected with LPS revealed a significant increase in oxidative stress observed in the levels of lipid peroxidation product malondialdehyde (MDA). Total antioxidant capacity (TAC) was diminished (Figures 12 and 13). Our results suggest that LPS-induced oxidative damage can be alleviated by the pretreatment with the *G. oblongata* marine extract and the effect is shown presumably by scavenging of the ROS by β-carotene among other bioactive secondary metabolites as an antioxidant by modulating the antioxidants status and lipid peroxidation by decreasing oxidative stress and increases the total antioxidant capacity, attenuating and protecting against LPS-induced inflammation as well as apoptosis [71,72]. In general, inflammatory activity is accompanied by inducible nitric oxide synthase, leading to the production of nitric oxide, which enhances the catalytic activity of COX2 via formation of the peroxinitrite anion. Kawata et al. [68] showed that carotenoids have the ability to inhibit the effect of LPS-induced *Cox2* and *Nos2* mRNA expression in RAW264.7 cells with low concentration (25 μM). One of the most important class of marine secondary metabolites identified by GC-MS in *G. oblongata* marine extract was vitamin A metabolite retinoic acid (RA) (Table 1) with its applications in the pharmaceutical and biomedical industries due to its biological activities in organogenesis, differentiation, as well as reproduction, a key role in mucosal immune responses and cell growth. RA plays also an important role in gene transcription and controlling the inflammatory diseases not only in the intestine, but also in other tissues. It is also a control factor for regulatory T cells and maintains its homeostasis [73,74]. In addition, due to its regulatory activity, retinoic acid has been shown to play an important role in the control of inflammatory diseases not only in the intestine [75], but also in other tissues, such as the central nervous system [76] and pulmonary mucosa [73]. Furthermore, RA have important role in the immune system one of which is, to maintain epithelial and mucosal homeostasis and have anti-inflammatory role. The potential effect of RA as anti-inflammatory is via decreasing the inflammatory processes, enhancing homeostasis and ameliorating harmful inflammatory responses in tissues and mucosa. RA is essential for the intestinal tolerance, and that through inducing IL-10, IL-22, Treg, cytokines and antimicrobial peptide (AMP) production, which lead to the inhibition of Th17. RA ameliorating airway inflammatory diseases such as rhinitis and asthma through inhibiting Th2/Th17 response and promoting Treg cells. Retinoids enhance and elevate TGF-β and type I collagen, decreasing MMPs (matrix metalloproteinase) in photoaging (AGE), and decrease IL-1 family cytokines and PSO (epidermal hyperplasia in psoriatic lesions). Additionally, RA can inhibit the expression as well as synthesis of inflammatory mediators (cytokines and chemokines), suppressing the inflammatory responses enhancing by obesity. Current study reported the presence of Folic acids, in GC-MS profiling previous studies, showed various biological activities. For example, Fatahi et al. [77] reported that folic acid supplementation could significantly decrease the serum C-reactive protein in women, patients with T2DM, and those with less than 12-week intervention

The *in vitro* present study was investigate the antioxidant capacity comparing with vitamin c that consider as the most commonly used vitamin. It is a vitamin that participates in animals in many biochemical processes. It is highly soluble in water and acts as an important antioxidants agent. Vitamin c is chemically capable of interacting with and functions as a known hydrosoluble antioxidant, with most of the physiologically relevant radicals and oxidants. Vitamin plays a significant function in the defense of different tissues against oxidative stress. Another important biological function of ascorbate is to serve as a co-substrate for several enzymes of hydroxylase and oxygenase, such as prolyl and lysyl hydroxylase, dopamine β-hydroxylase, ascorbate peroxidase, and cytochrome b561 (Cyt b561), while maintaining their active centre metal ions for optimal enzyme activity in a reduced state (as an electron donor) [87].

## Conclusion

This present study demonstrated that the *G. oblongata* marine extract had protective effects against acute liver and kidney injuries in mice via alleviating inflammatory response and oxidative stress burden through lowering serum cytokines, including NFkB, MPO and LPO, and improving liver apoptosis through suppressing protein tyrosine kinase signaling pathway. These findings revealed a detrimental role of the *G. oblongata* marine extract in LPS-induced inflammation and organ injuries, and that may be due to antioxidant activity. These data suggested that the *G. oblongata* marine extract source for biotechnological, nutraceutical and pharmaceutical applications that most possibly act as promising therapeutic agents for LPS-induced acute liver and kidney injuries through antioxidant and anti-inflammatory pathways.

## Data Availability

All data are including in the paper.

## Abbreviations

ABTS: 2,2′-azino-bis-3-ethylbenzthiazoline-6-sulphonic acid
ALI: acute liver injury
ALT: alanine aminotransferase
AMP: antimicrobial peptide
AST: aspartate aminotransferase
CRP: C-reactive protein
CYP: cytochrome P450
CYP2E1: cytochrome P450 2E1:
DHA: docosahexaenoic acid
EPA: eicosapentaenoic acid
GC-MS: gas chromatography–mass spectrometry
H_2_O_2_: hydrogen peroxide
IκBα: nuclear factor of kappa light polypeptide gene enhancer in B-cells inhibitor, alpha
IL-10: interleukin 10
IL-22: interleukin 22
LPO: lipid peroxidation
LPS: lipopolysaccharide
MDA: malondialdehyde
MMP: matrix metalloproteinase
MPO: myeloperoxidase
mRNA: messenger RNA
n-3 PUFA: *n*-3 polyunsaturated fatty acid
NADPH: nicotinamide adenine dinucleotide phosphate
NF-κB: nuclear factor-kappaB
NO: nitric oxide
NOS2: nitric oxide synthase
p65: transcription factor p65 (RELA)
PSO: epidermal hyperplasia in psoriatic lesions
PTK: protein tyrosine kinase
RA: retinoic acid
ROS: reactive oxygen species
T2DM: diabetics type 2
TAC: total antioxidant capacity
TGF-β: transforming growth factor beta
Th2/Th17: T helper type 2
TLR4: Toll-like receptor 4
TMB: 3, 3′, 5, 5′-tetramethylbenzidine
TNF-α: tumor necrosis factor
Treg: the regulatory T cells
VitC: vitamin C
