# Correction: Red Algae Natural Products for Prevention of Lipopolysaccharides (LPS)-induced liver and kidney inflammation and Injuries

**DOI:** 10.1042/BSR-2020-2022_COR

**Published:** 2021-10-18

**Keywords:** G. Oblongata red algae, Natural products, LPS, Liver, Kidney, Inflammation

The abstract of the original article “Red Algae Natural Products for Prevention of Lipopolysaccharides (LPS)-induced liver and kidney inflammation and Injuries” (*Biosci Rep* (2021) **41**(1), https://doi.org/10.1042/BSR20202022) had been published with an error that had been introduced at an early editing stage. The name ‘*G. oblongata*’ should have been expanded to ‘*Galaxaura oblongata*’ rather than ‘*Gromphadorhina oblongata*’ as was stated on the Version of Record. This typographical error was updated on the original article on 18 October 2021.

